# A design to improve the comparability of area maps: the example of the premature mortality in Belgium

**DOI:** 10.1186/2049-3258-73-S1-P32

**Published:** 2015-09-17

**Authors:** Francoise Renard, Patrick Deboosere, Jean Tafforeau

**Affiliations:** 1 The Scientific Institute of Public Health WIV-ISP, Brussels, Belgium; 2 Vrije Universiteit, Brussels, Belgium

## Methods

A diverging green-to-red colour scale is used, where the cut-off colour (yellow) represents a reference value with the same meaning across all maps: the average sex-specific mortality rate for the cause of death studied. To represent the relative disparity, rates are classified according to a geometric progression with a 1.1 step, meaning that each colour class has a mortality rate 1.1 times higher than the preceding one. The number of classes is determined by the disparity between the extreme districts: the larger the disparity, the more colour classes present and the sharper the colour contrast. The relative distance between the highest and the lowest classes is 1.1 The midpoint of each class is calculated as the average rate * (1.1) rank of the class, starting from the average. The legend of the maps displays the boundaries of each class.

## Results

The all-causes premature mortality map displays 8 colour classes in males and 6 in females, expressing a stronger between-district disparity in men (RR= 1.8) than in women (RR=1.6). The highest class (observed in Mons and Charleroi) ranks at the 4^th^ position above the average (being 490.6 per 100.000). The midpoint of this highest class is calculated as 490.6*(1.1^4^) = 718 per 100.000. The lowest rate is observed in Maaseik (3^rd^ class below the average). The midpoint of this lowest class is 490.6*(1.1^-3^) = 368.5 per 100.000.

## Conclusion

the use of a relative scale allows an easy comparison between maps and shows at a glance the relative between-district disparity.

